# Updated pathology reporting standards for bladder cancer: biopsies, transurethral resections and radical cystectomies

**DOI:** 10.1007/s00345-021-03831-1

**Published:** 2021-09-23

**Authors:** Eva Compérat, André Oszwald, Gabriel Wasinger, Donna E. Hansel, Rodolfo Montironi, Theodorus van der Kwast, Johannes A. Witjes, Mahul B. Amin

**Affiliations:** 1 Department of Pathology, Hôpital Tenon, Sorbonne University, Paris VI, Paris, France; 2 Department of Pathology, General Hospital, Vienna Medical University, Vienna, Austria; 3 Department of Pathology and Laboratory Medicine, School of Medicine, Oregon Health and Science University, Portland, OR USA; 4 Department of Pathology, School of Medicine, United Hospitals, Polytechnic University of the Marche Region, Ancona, Italy; 5 Department of Pathology, Princess Margaret Cancer Center, University Health Network, University of Toronto, Toronto, ON Canada; 6 Department of Urology, Radboudumc, Nijmegen, The Netherlands; 7 Department of Pathology and Laboratory Medicine and Urology, University of Tennessee Health Science, Memphis, TN USA

**Keywords:** Bladder cancer, Pathology, Reporting, Staging, Cystectomy, TURB

## Abstract

**Aim:**

Optimal management of bladder cancer requires an accurate, standardised and timely pathological diagnosis, and close communication between surgeons and pathologists. Here, we provide an update on pathology reporting standards of transurethral resections of the bladder and cystectomies.

**Methods:**

We reviewed recent literature, focusing on developments between 2013 and 2021.

**Results:**

Published reporting standards developed by pathology organizations have improved diagnosis and treatment. Tumor sub-staging and subtyping has gained increased attention. Lymph nodes continue to be an area of debate, and their staging has seen minor modifications. Several tasks, particularly regarding specimen preparation (“grossing”), are not yet standardized and offer opportunity for improvement. Molecular classification is rapidly evolving, but currently has only limited impact on management.

**Conclusion:**

Pathological reporting of bladder cancer is continuously evolving and remains challenging in some areas. This review provides an overview of recent major developments, with a particular focus on published reporting standards.

## Introduction

Over the past years, pathology organizations have developed best practices and reporting standards which allow pathologists to report with greater conformity and interobserver agreement. One important milestone in genitourinary pathology was the 2005 modified Gleason score, but strict reporting standards have also been applied to other fields such as bladder cancer (BC). Following the groundwork laid by the WHO 2016, such standards are continually revised by the College of American Pathologists (CAP) and the International Collaboration on Cancer Reporting (ICCR), and are accessible to pathologists worldwide online [1–3].

Reporting standards have been integrated into guidelines such as the EAU guidelines on non-muscle invasive bladder cancer (NMIBC) and muscle invasive bladder cancer (MIBC), and aid urologists in determining if reporting standards are followed by their colleague pathologists [4]. These efforts foster a multidisciplinary approach involving pathologists, radiologists, urologists and oncologists, especially in the context of tumor boards, necessary to achieve the optimal management for patients.

Despite established standards, reporting the spectrum of bladder cancer pathology, key for appropriate treatment, remains challenging; in particular, the evaluation of biopsies and TURB (transurethral resections of the bladder) can be extremely difficult for several reasons, which we will explain in detail here.

Importantly, several tasks are not yet standardised (e.g., the number of tissue blocks produced from a large TURB, or grossing of cystectomy and lymphadenectomy specimens), for which we provide recommendations based on our current practices.

## Acquisition of data

In this paper, we reviewed recent international literature and reviewed recent relevant studies on reporting of BCa, in particular regarding: grossing, biopsies and TURB, cystectomies, staging, substaging and grading, with an emphasis on developments between 2013 and 2021.

## Communication between urologists and pathologists

Besides consistent reporting based on established guidelines, specialist review may change more than a third of diagnoses with therapeutic implications in T1–T2 bladder cancer patients [4]. Histological subtypes of bladder cancer have significant impact on treatment decisions, and both underreporting and overreporting remain issues with general pathology reads.

The urologist can greatly aid high-quality analysis by providing adequate information on the submitted tissue. This includes clinical information (current presentation and disease history including local and systemic therapies, e.g. chemotherapy or pelvic radiation), information on the anatomic location (e.g. prostatic urethra, trigone) and other lesional characteristics (papillary, sessile tumor).

In case of MIBC and radical surgery, the pathologist should be informed of neoadjuvant chemo-, immuno- or radiation therapy, which impacts the composition and architecture of both normal and tumour bladder and lymph node tissue. Stitches or clips with a clear description may be useful for sample orientation. It is important that the surgeon should not open the bladder after resection, or do it with the pathologist to not jeopardize the assessment of resection margins.

## Reporting of biopsies and TURB

Although much information is obtained from TURB/biopsies, the limitations must be appreciated. For example, guidelines recommend not to designate a higher stage than T2. Substaging (T2a, inner detrusor layer versus T2b, outer detrusor layer) should not be performed in a TURB/biopsy specimen, as this determination can only be reliably preformed in a cystectomy.

One frequent misunderstanding arises from the presence of adipose tissue in a specimen, which is present not only in the perivesicular fat tissue, but also in the lamina propria or the normal detrusor muscle, frequently in neurogenic or trabecular bladders. Hence, presence of tumor in adipose tissue does not indicate a T3 tumor in a TURB specimen.

Data regarding assessment of residual tumor in TURB/biopsy are limited. The EAU supports submitting the base and deep part of the resection in separate containers. En bloc resections can sometimes be inked on resection margins if the sample is sent in an oriented manner and in one piece.

### T1 substaging

The WHO (2016) encourages T1 substaging by depth and/or extent of subepithelial tissue invasion without specifying a method, and argues that “details on how to do so are yet to be agreed upon” [5]. Recently, the Genitourinary Pathology Society has highlighted the advantages and disadvantages of histoanatomic and micrometric approaches, yet did not favour a specific method. Instead, they highlighted the recommendation to perform substaging when possible, and the need for consistency through standardisation of TUR processing and measurement procedures [6].

### En bloc resections (EBR)

A recent randomized study showed that EBR is clinically safe, especially with the submucosal hydrodissection technique, and significantly improved histopathological assessment concerning musclaris propria (MP) invasion [7]. In another study, EBR provided high-quality specimens for determining invasion of muscularis mucosae (MM), where deeply invading T1 BC demonstrated poor prognosis [8].

It is noteworthy that EBR specimens may be very small, and the amount of MP may be limited or absent. Sampling and reporting protocols for margins are not standardized and best performed in individual cases based on good communication between the pathologist and the urologist.

## Reporting of cystectomies

**Fig. 1:**
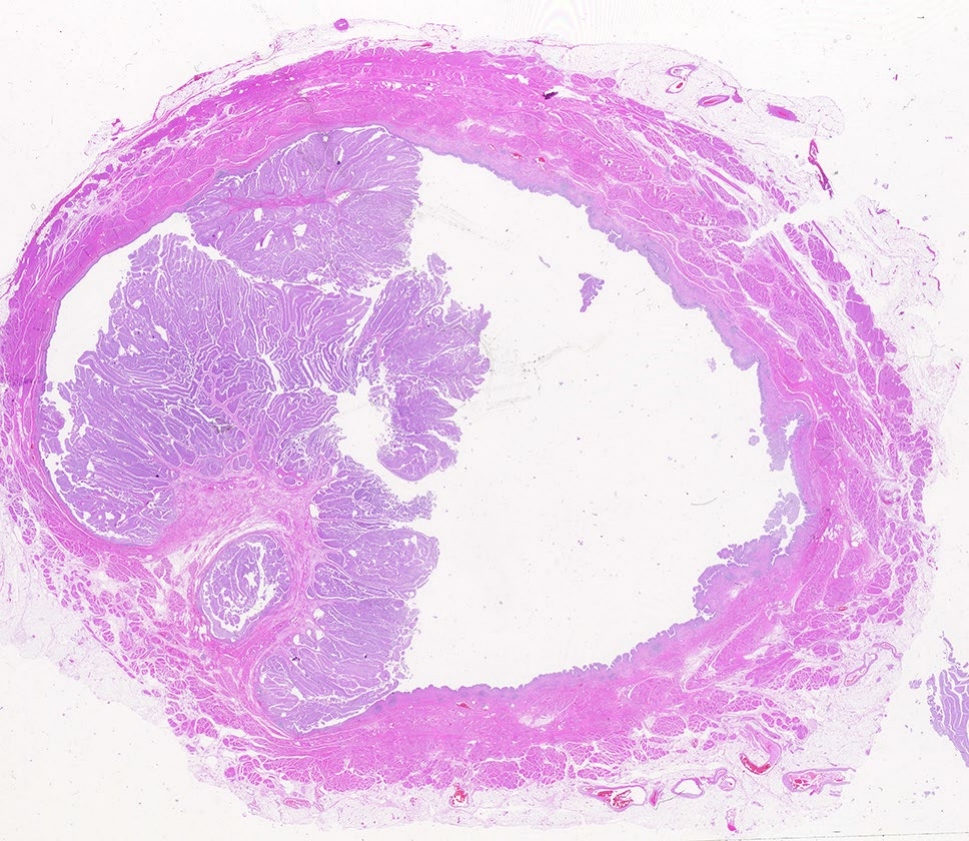
Whole mount section of a cystectomy specimen with a superficial papillary tumor (pTa), without invasion into the lamina propria or muscularis propria

There is opportunity for more standardization of grossing of RC specimens (Fig. 1) especially with respect to the number and location of the Sections [9]. The International Collaboration on Cancer Reporting (ICCR) and the College of American Pathologists (CAP) have given recommendations which have already been discussed above [2]. In the author’s experience, macroscopically evident lesions such as scars or red plaques (which can be CIS) are best visible and may be inked in the fresh cystectomy specimen, since their identification tends to become more difficult after fixation. The CAP has provided recommendations on the selection of samples for microscopic evaluation to facilitate adequate evaluation of invasion depth, adjacent tissues and resection margins [1].

### Staging

Accurate substaging of pT2 disease is relevant for prognosis of MIBC. A study of patients with pT2N0 disease showed worse prognosis in pT2b patients (5-year RFS: 85.9% vs 37.5%, 5-year CSS: 84.8% vs 59.6%. Furthermore, proportional hazards regression showed that pT2 substaging was the only independent risk factor of recurrence and cancer-specific death [10]. Thus, at cystectomy it is highly relevant to substratify pT2N0 UC.

Assessment of perivesical fat invasion (pT2 vs pT3a/b) can be challenging and leads to some degree of interobserver variability, even among experts [11], due to the poorly demarcated junction between the perivesical fat and the outer layer of the MP, which typically presents as haphazardly separated muscle bundles.

**Fig. 2:**
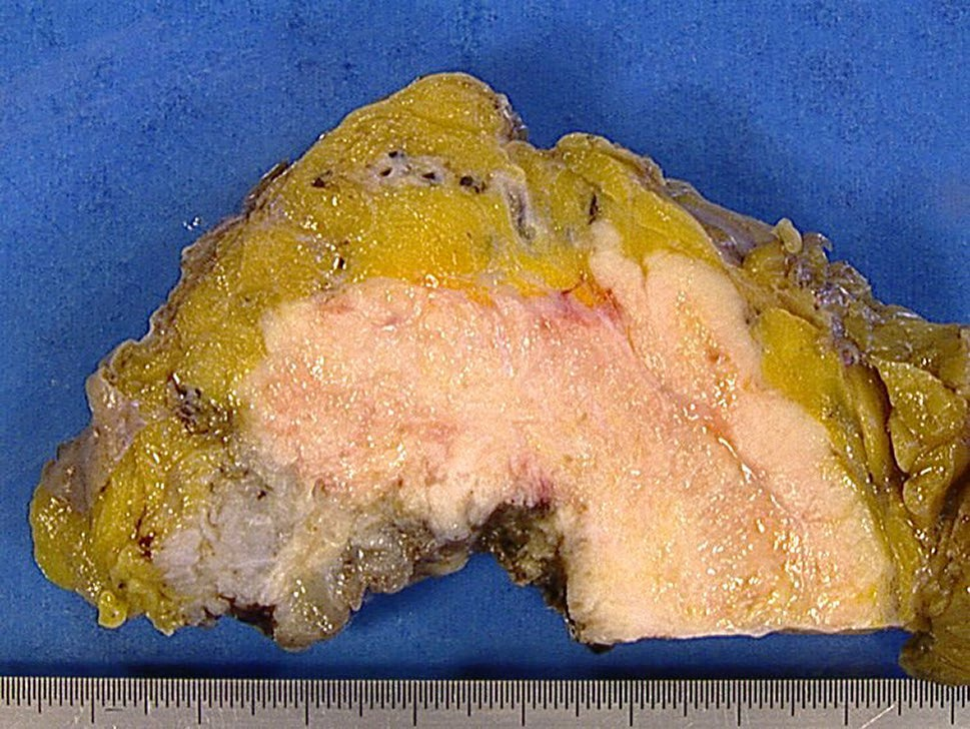
Gross aspect of pT3b bladder cancer. The growth of the tumor (white) from the bladder lumen (bottom) into the perivesicular adipose tissue (top, yellow) is macroscopically visible and, therefore, qualifies as pT3b

Determination of perivesical fat invasion may be compounded by extensive desmoplasia and fibrosis surrounding an invasive carcinoma and requires generous sampling and close examination. Tumors with grossly visible fat invasion are considered pT3b (Fig. 2) and are prognostically distinct from pT3a. Invasion into adjacent structures is designated pT4. In contrast to prostatic invasion via the bladder wall or the perivesical fat (pT4), subepithelial or stromal invasion of the prostate via the urethra is staged via the urethral staging system and is designated pT1 or pT2, respectively [12].

### NAC

NAC has become a standard treatment for patients to undergo cystectomy for ≥ T2 UC, and the pathological response to NAC is prognostic. Patients with complete response (designated ypT0 by UICC and AJCC 8th ed.), or down-staging to lower than pT2N0 have a 5-year overall survival (OS) of 85% in contrast to approximately 30% to 40% for those without evidence of tumor down-staging [13]. A study of 165 patients with invasive UC and RC after NAC investigated a tumor regression grade, including therapy-related stromal and epithelial changes, most commonly fibrotic stroma and poorly preserved chromatin in remaining UC epithelium. Despite frequent stromal (41%) and epithelial (5%) changes post-NAC, multivariate analysis showed that only pTN stage and margin status, but not tumor regression grade, predicted progression and cancer related death. Thus, the “traditional” histological parameters in RC remain the best predictors of disease course post-NAC [14].

A recent study of patients with pN1-3 disease at time of RC found that among 450 patients who received platinum-based NAC, that the number of positive lymph nodes independently predicted OS (*p* = 0.013). Patients with persistent nodal disease in post-NAC had worse prognosis than those with nodal disease after upfront RC.

### Diverticula

Acquired diverticula may still contain sparse detrusor muscle, while congenital diverticula are absent detrusor muscle in the affected portion of the bladder wall. Epithelial neoplasms have been reported in up to 14% of bladder diverticula, contributing approximately 1% of bladder neoplasms [15]. In case of absent detrusor muscle, respective tumors cannot be designated T2 stage, and pathologists can report only Ta-1 and T3–4 stages.

Recently, a study described a fraction of cystectomy patients (exclusively male) who had UC in diverticula, about half of which presented with their highest tumor stage in the diverticula, and higher rates of upstaging upon RC (48 vs 39%). In multivariate analysis, UC in bladder diverticula was not independently associated with significantly different recurrence-free survival or overall survival [16]. Some patients may benefit from bladder-sparing partial cystectomy; Voskuilen et al. recently retrospectively analyzed patients with UC in diverticula treated either by RC (*n* = 81) or by partial cystectomy (*n* = 34), and found no significant difference in 5-year OS or metastasis free survival (62% vs 66% and 66% vs 55%, respectively) [17].

### Margins

Surgical margins submitted for histology per protocol include ureters, urethra, perivesical soft tissue in RC and bladder wall margins in partial cystectomy. Additional sections should be submitted where there is gross suspicion for positive margins. Studies have reported positive surgical margins in up to 15% of RC specimens, typically at the urethra, the ureters or the soft tissue. While positive urethral/ureteral margins usually show carcinoma in situ (Cis), positive soft tissue margins are (which are associated with an adverse outcome) mostly show invasive UC. A French multi-institutional case–control study reported a significantly higher recurrence rate and decreased cancer-specific survival for patients with positive urethral and soft tissue, but not ureteral margins [18]. In multivariate analysis, both urethral and soft tissue margins were independent factors for recurrence, but only soft tissue margins affected cancer-specific survival. It is worth noting that some tumor subtypes (i.e. plasmacytoid) very frequently have positive margins upon resection (see section on subtypes).

### Carcinoma in situ (CIS)

A recent meta-analysis found that concomitant CIS, a known prognostic factor [19], was reported in 39.4% of radical cystectomy specimens [20]. In analyses including all patients, concomitant CIS was associated with ureteral involvement, but not with significant differences in mortality or recurrence-free survival. On sub-analysis of studies restricted to patients with organ confined bladder cancer at RC, concomitant CIS was associated with worse recurrence-free survival and greater cancer-specific mortality. Urine cytology is an important diagnostic tool in the management of CIS, as it boasts high sensitivity and specificity for high-grade lesions.

### Frozen sections

Frozen sections of the ureteral margins at cystectomy are a reliable examination with a sensitivity and specificity of approximately 75% and 99%, and are positive in around 9% of cases [21]. Nevertheless, the EAU guidelines do not recommend their routine use [4]. Since CIS has a known propensity for multifocality, lesions proximal or distal to the section may be missed. Frozen sections on the urethral margin are also feasible, although often (especially in cases with reconstruction of an orthotopic neobladder), a prior biopsy is sufficient to determine the presence of CIS. Specimens for frozen sections must be sent orientated by clear marking of the actual surgical margin to facilitate accurate examination.

### Lymph node status

In recent years, several studies have been performed regarding both surgical and pathological aspects of lymph node dissection (LND). The current edition of the AJCC staging manual [12] distinguishes N0 (no LN metastasis) from N1 and N2 (single or multiple regional LN metastasis in the true pelvis) from N3 (metastasis to the common iliac LN). Importantly, perivesical lymph nodes are now considered regional lymph nodes as well and their metastatic condition implies pN1. LN metastases outside of these regions are considered distant metastasis and designated M1a, which considerably changes the patient’s management.

The ideal extent of LND is currently under debate. To facilitate comparison between studies, the EAU has provided a standardized terminology for LND: limited (obturator and perivesical fossa), standard (including common iliac arteries), extended (up to the aortic bifurcation, with or without pre-sacral LN), and super-extended (up to the inferior mesenteric artery). The authors concluded in their review that any LND is better than no LND [22].

### Issues with LND from the pathologist’s perspective

The gross examination of resected tissue is a central component of the pelvic lymph node dissection (PLND) assessment. Currently, there is no consensus on the optimal handling of PLND specimens.

In practice, all palpable lymph nodes (LN) or firm tissue should be submitted to ensure a thorough examination. Pelvic LN in particular frequently show adipose change and tortuous configuration, and careful dissection as well as adherence to strict histological criteria is essential for accurate enumeration [23]. It has been shown that en bloc submission of lymph node dissection (LND) yields lower total but similar positive counts of LN [24].

The lymph node density, the ratio of positive to total removed LN, has been validated and thus confirms the recommendation to report total and positive numbers of lymph nodes [25]. However, it places a critical task on the pathologist who must accurately count total lymph nodes, which is highly variable between observers. A survey of ten pathologists identified areas of considerable disagreement between pathologists in microscopic LN assessment: the smallest structures eligible for counting, the separation of spatially related structures, and the conflict of gross vs. microscopic enumeration [26]. Similarly, a survey by the European Network of Uropathology highlighted the substantial variation in assessment of PLND in 23 countries, for instance in rate of serial sections and reporting practice [27].

Few data exist about bladder cancer, but regarding prostate cancer, Engvad et al. reported that serial sectioning of LN resulted in upstaging of 2.3% of patients [28]. Current routine processing of samples can never guarantee an absolute identification of positive submitted LN. Nonetheless, there is currently no recommendation for extensive sectioning or routine immunohistochemistry (IHC) staining.

In summary, the handling practices for surgery, grossing and microscopic examination of PLND specimens are as of yet unresolved challenges and will need to be addressed in a consensus of urologists and pathologists. Future research may help to delineate which information is necessary for surgeons and oncologists to be able to provide the best possible stratification and a tailored approach to therapy.

## General aspects

### Lymphovascular invasion

Lymphovascular invasion (LVI) is an important step in bladder cancer cell dissemination, and, therefore, mandatory to report in biopsies, TURBs and RCs. A meta-analysis of 65 studies (including 78,107 patients) found that LVI was reported in 35.4% of patients and was associated with disease recurrence ([HR] = 1.57) and cancer-specific mortality (HR = 1.59) regardless of tumor stage and node status. Therefore, LVI should be part of all UC reporting, especially in T1 tumours, and could provide additional information for treatment decisions regarding adjuvant therapy after RC [29]. LVI is often overdiagnosed by pathologists because retraction artifact, common in bladder cancers, may mimic LVI. Nonetheless, it is not recommended to perform routine IHC to aid its detection due to the disproportional cost and delay of reports [30].

### Grading

According to the WHO 2016 and the NMIBC guidelines 2021 the grading of a tumor is important and shall be done according to the WHO 2016 system. The current 2-tiered grading system—high versus low grade—is intended to simplify clinical decision making in daily practice over the 3-tiered 1973 system. It also provides congruence between histology and cytology reports, and highlights the prompt therapeutic requirement for all high-grade lesions (flat or papillary) [31]. A meta-analysis of 20 studies concluded that the new system does not outperform the 1973 system in prognostic value, but shows higher reproducibility [32]. Some authors prefer to provide tumor grades according to both 2016 and 1973 systems, but neither WHO nor most medical associations endorse this practice. Since the vast majority of invasive tumors (> pT1) are high grade according to the 2016 grading system, it does not accommodate further stratification for these tumors by grade. However, the WHO systems 2016 (and 2020) state that stratification of invasive tumors is important and shall be performed by substaging, for which there exist defined criteria and methods.

### ICCR standards for TURB/biopsy and cystectomy

The ICCR recently published standards for reporting biopsies and TURB, as well as cystectomies. Items have been designated as required (i.e. mandatory) or recommended as follows (Table 1):

**Table 1:** Required and recommended features to report in bladder specimens, according to the current ICCR dataset [2, 3] (www.ICCR-cancer.org)

	Status
Clinical information (e.g. previous therapy)	Recommended
Specimen site	*Required*
Operative procedure	*Required*
Block identification key (TURB)	Recommended
Histological tumor type	*Required*
Presence of invasive carcinoma	*Required*
Associated epithelial lesions	Recommended
Histological grade	*Required*
Extent of invasion	*Required*
Macroscopic extent of invasion	*Required*
Microscopic extent of invasion	*Required*
Tumor focality	Recommended
Substaging T1 disease	Recommended
Lymphovascular invasion	*Required*
*Cystectomy only*:	
Response to neoadjuvant therapy	*Required*
Margin status	*Required*
Lymph node status	*Required*
Histologically confirmed metastasis	*Required*
Coexistent pathology	Recommended
Histological staging (if applicable)	Recommended

### Artifacts that influence pathologic staging

Proper pathology reporting is extremely dependent on the quality of the submitted material. Cautery artifact may hinder accurate staging at initial transurethral resection of bladder (TURB) for large tumors by understaging up to 6% of patients [33]. A recent study underlines that TURB is a critical step in the management of bladder cancer; therefore, training of young urologists to acquire necessary technical skills to perform adequate TURB or biopsy should be a priority [34]. From a clinical point of view, bipolar TURB is advantageous in terms of operation and hospitalization time [35], while from the pathologists’ point of view, bipolar TURB results in less tissue artifacts [36]. An improved resection of the detrusor muscle sampling rate after bipolar TURB has been reported and positively affects correct staging [37].

Other sources of artifacts, such as tangential sections, tissue fragmentation and necrosis remain more in the domain of pathology and are not urologist-related.

### Up- and down-staging

Several authors have highlighted the frequent up- or down-staging of UC in biopsies and TURBs due to interobserver variation among pathologists. A publication by several authors of this review showed that in difficult cases full agreement was only obtained in 44% of cases with a multi-rater kappa score of 0.47 [38]. Indeed, pathologists must be cautious in several situations: One challenge is the different structure of the bladder according to the anatomic location, where TURB was performed. The muscularis mucosae is often not identifiable at the trigone, the detrusor muscle on the other hand can be extremely thick in this area because of the insertion of the ureters [39]. Therefore, lack of information on biopsy or TURB location may influence staging outcome. The differentiation of muscularis mucosae and muscularis propria is also often considered a common challenge in certain situations [40]. Several immunohistochemical markers have been proposed and are variably used distinguish muscularis mucosae and muscularis propria, but as for now, no single marker can reliably differentiate between them and any use must be in the context of strict histological correlation [41]. Correct staging is also often challenging after multiple resections, which leads to hypertrophy, different orientation, or even desmoplastic reaction of lamina propria and/or muscularis mucosae. Other factors which might influence staging are thermal or crush artifacts, as discussed before, as well as necrosis, especially if extensive.

In cystectomies staging is easier, although the staging of pT2b (deep muscle invasion) versus pT3a (fat invasion) tumors may be problematic as there is no often no clear histological distinction between the two, especially if the tumor is surrounded by desmoplastic stroma [11, 42].

### Predictors of BCG response

Bacillus Calmette–Guérin (BCG) is currently the most effective intravesical therapy for non-muscle invasive bladder cancer (NMIBC), reducing not only recurrence rates but also stage progression and deaths [43].

Although there are no good histological predictors, several studies have provided potential alternatives to predict BCG response: A recent study with 50 patients showed some promise of a panel of urine cytokines measured at varying time points. Among clinicopathologic variables, history of tobacco smoking was associated with an improved response rate (HR 0.38; *P* = 0.04). The dynamics of urinary IL18-binding protein-a (HR 1.995; *P* = 0.01), IL23 (HR 1.12), IL8 (HR 0.27; 95% CI 0.07–1.08; *P* = 0.06), and IFNγ-induced protein-10 (HR 0.95; 95% CI 0.91–0.99; *P* = 0.04) at week 13 from baseline were the best predictors of response to BCG therapy in NMIBC [44]. A recent systematic review allowed to give some insights into several options for predicting BCG response [45], showing that some risk nomograms revealed clinicopathologic features, especially tumor stage and grade, as the most effective predictors of BCG response, which underlines the important role of pathology in BCG treatment. Data are less robust in regards to the association of response with age, sex, recurrent tumors, multiplicity of tumors, and the presence of carcinoma in situ (CIS). Some biomarkers, such as tumor p53 and urinary interleukin-2 expression, had only limited success in predicting BCG response, possibly due to the multifaceted nature of the generated immune response. Gene expression data correlate with disease progression, but studies examining potential associations with BCG response are limited. Recent trials focused on patients with CIS unresponsive to BCG and led to the recent FDA approval of pembrolizumab for this indication, though more data are still required [45–47]. On the other hand, as for now, no tissue marker has been recommended for routine to predict responsiveness to BCG after first TURB.

### Urothelial carcinoma subtypes, divergent differentiation and relation to molecular classification

Invasive urothelial carcinoma has a remarkable propensity for morphological diversity due to divergent differentiation and histological subtypes. Much literature has been devoted to the characterization and definition of histological entities, but only few prospective data exist [48]. Recently, molecular classification (i.e. on basis of expression and genetic alterations) has enriched our understanding of bladder cancer and provided us with a new framework for stratification and assessing response to different therapy regimens [49]. It is important to understand that when talking about divergent differentiation or subtypes, a therapeutic implication exists. Therefore, the pathologist must be aware of the diagnostic criteria and accurately report them.

### Tumor type

According to WHO, CAP and ICCR guidelines [1–3, 5], the presence of any urothelial component (including pTis) within a malignant lesion invokes the diagnosis of urothelial carcinoma. As notable exception, any quantity of neuroendocrine component yields the diagnosis of a neuroendocrine tumor and dictates its according management. Only “pure” squamous cell carcinoma (SCC), adenocarcinoma (AC), or Mullerian carcinoma should be designated as such. Urachal carcinomas are diagnosed only on the basis of strict clinicopathologic correlation and not on histology alone (lesion located within, and absence of diffuse intestinal metaplasia/cystitis glandularis outside of, bladder dome or anterior wall, epicenter in bladder wall or perivesical tissue, absence of known primary elsewhere) [50].

### Subtypes (formerly variants) and divergent differentiation

Like previous editions, the 4th edition of the WHO classification (2016) [5] recognizes the morphological diversity of urothelial carcinoma. A component that differs from the nondescript histological appearance of most urothelial cancers (“pure” urothelial carcinoma) is termed a histological subtype from the WHO 2021 onward (formerly “variant histology”). Moreover, the WHO 2016 designates some forms with the term “divergent differentiation” (squamous, glandular, trophoblastic). This distinction exists to reflect the circumstance that the appearance of divergent differentiation is arguably similar to what can be observed in other non-tumoral epithelial tissue, while specific histologic-subtypes are morphological patterns that are virtually exclusive to neoplastic disease.

The presence of subtypes and/or divergent differentiation is important for several reasons: first, reporting the relative amount of subtypes (as percentage) in a sample, as already recommended by WHO 2016, CAP and ICCR, enables clinicians to treat patients according to the latest results of the literature regarding prognostic or therapeutic implications. The prevalence of subtypes in TURB specimens is likely underreported, especially in community practice [51] and might, therefore, be insufficient to evaluate subtype presence [52]. Second, from the pathologists’ perspective, some subtypes deserve special attention since they are diagnostic “pitfalls” in that they exhibit a high frequency of understaging or altogether risk misperception for a benign or low-grade lesion, or require a differential diagnosis including metastatic disease originating in another organ [53]. Lastly, it enables more accurate data on their prevalence, which in turn may help understanding their biology and their clinical consequences.

If more than one subtype is present, documentation of percentage of each is recommended. Supporting data regarding the significance of percentage reporting of a given subtype is limited. Some studies especially tried to review this for micropapillary, sarcomatoid, lymphoepithelioma-like subtypes and carcinomas with divergent differentiation (glandular, squamous). Nevertheless, data are not robust, well-defined cut points are not yet available and most of these studies are limited in numbers and retrospective design [1, 2, 5, 54].

A 2019 review of the literature by the EAU panel concluded that data on prognosis and treatment of UC subtypes are immature and heterogeneous, and that all patients with MIBC should be treated with RC [48]. While mixed histology tumors are treated analogue to pure urothelial tumors, there is no evidence for a role of NACT in pure squamous or adenocarcinomas, in contrast to neuroendocrine tumors which should all receive NACT [55].

Due to the heterogeneity in data quality and the constantly shifting ground of our knowledge, it is generally recommended to report all morphological aspects of bladder cancer. Specifically, for both TURB and RC, the WHO, CAP and ICCR recommend reporting the presence and percentage of any subtype [2, 3] or differentiation present in a urinary bladder specimen, and the percentage of squamous, glandular, trophoblastic, Müllerian and neuroendocrine differentiation [1].

Some subtypes and UC with divergent differentiation are briefly discussed due to their frequent presence or due to recent updates:

*Squamous cell* differentiation is the most common histological pattern in up to 40% of tumors [56]. While the distinction is usually straightforward, these cancers stain positively for CK5/6 and CK5/14 [57]. When considering the molecular underpinnings, KRT 5/6 and 14 are overrepresented in the squamous/basal molecular class [49].

*Glandular* differentiation—characterized by true gland formation—has previously been reported in up to 18% of invasive tumors [56]. A recent report shows that in pT1 tumors, glandular differentiation predicts poor prognosis [58], in contrast to histology at RC [59]. It is unclear if distinction of UC with glandular differentiation from “pure” UC of the bladder has any clinical consequence.

*Neuroendocrine* differentiation (small-/large cell) is rare with < 1% of all bladder tumors [60]. Expression of neuroendocrine marker supports the primarily morphological diagnosis. Any neuroendocrine component renders a diagnosis of neuroendocrine tumor, and the portion of nondescript urothelial (or other) subtype histology is to be specified. In the recent molecular classification consensus, neuroendocrine tumors were almost exclusively present in the “neuroendocrine-like” class, which showed the poorest survival of all UC [49].

**Fig. 3:**
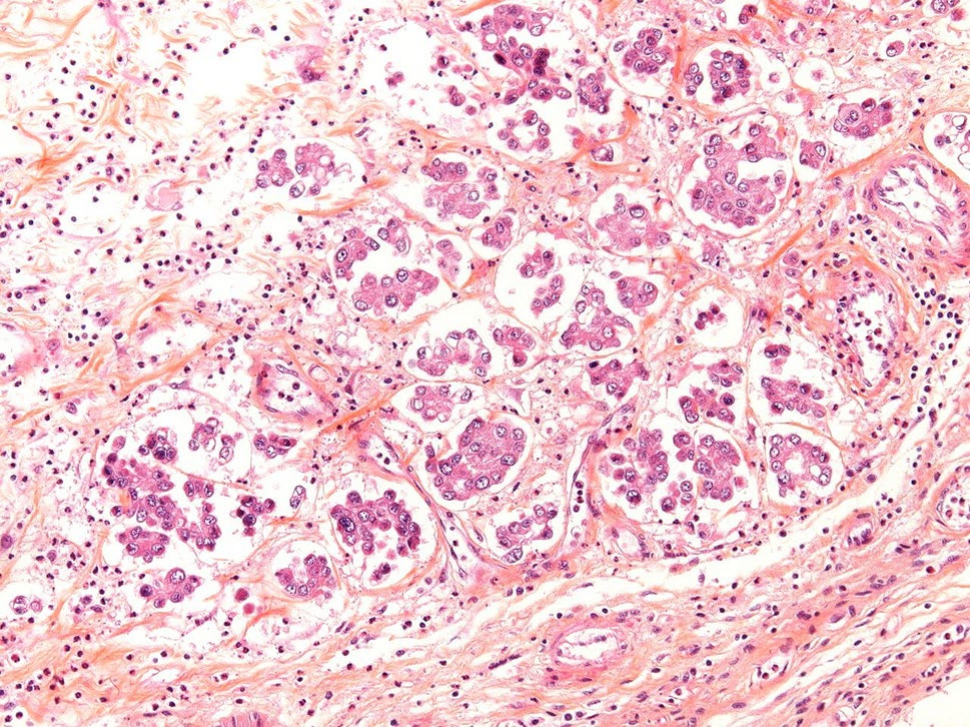
Typical micropapillary UC infiltrating the lamina propria. Retraction artefact around the tumor cells may mimic lymphovascular invasion. Hematoxylin-eosine-safranine stain

*Micropapillary UC* (MPUC) (Fig. 3) is attributed in the presence of micropapillary architecture, reminiscent of the configuration seen in ovarian papillary serous tumors [61]. The evidence regarding the oncological outcome of MPUC and benefit of neoadjuvant chemotherapy is conflicting [62–64].Transcriptomic analysis of 43 MPUC showed that they were almost all of the luminal subtype [65], see Table 2.

**Table 2:** Suggested molecular consensus groups according to Kamoun et al. [49], percentage in brackets specifies reported frequency

Molecular consensus groups	Corresponding histological aspects
Luminal papillary (24%)	Papillary aspects
Luminal non-specified (8%)	Papillary and micropapillary aspects, associated with carcinoma in situ
Luminal unstable (15%)	Papillary aspects (less than other luminal types)
Stroma-rich (15%)	Higher proportion of smooth muscle cells
Basal/squamous (35%)	Squamous differentiation
Neuroendocrine-like (3%)	Neuroendocrine carcinoma

**Fig. 4:**
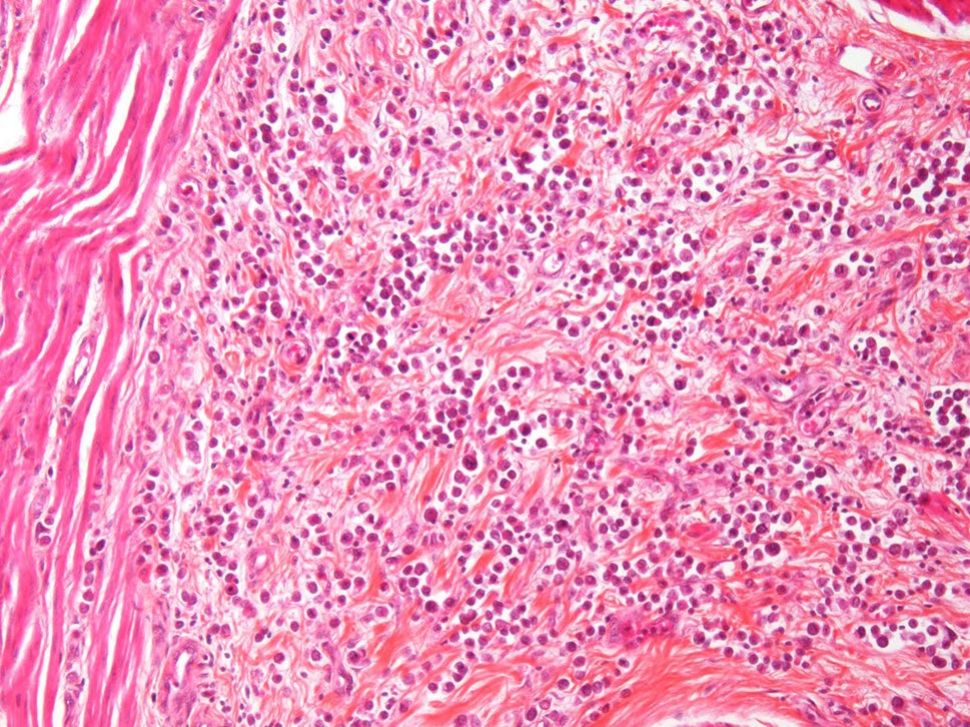
Plasmacytoid UC with discohesive tumor cells resembling plasma cells with infiltrative growth between connective tissue and muscle fibres. Hematoxylin-eosine-safranine stain

*The plasmacytoid* UC (Fig. 4) is very rare [66] and has potential surgical implications, since local control is challenging due to its reported invasion along fascial sheets [67, 68]. The data on treatment of plasmacytoid UC are based on small case series [68, 69] and the benefit of neoadjuvant chemotherapy is unclear.

*Nested and large nested* UC display nests of cells, characteristically with deceptively mild pleomorphism and only slightly increased nuclear-cytoplasmic ratio. Also in this subtype, there seem to be differences between pure and mixed forms; pure large nested carcinoma has been shown to have a predominantly luminal-papillary phenotype with higher rate of FGFR3 mutations than mixed large nested subtype [70].

### Subtypes (variants), divergent differentiation and molecular overlaps

Since 2012, evolving molecular classifications aim to offer more specific treatment for BC. Initially, authors claimed that these groups were independent of histological findings [71]. Later studies could show a partial overlap and a recent paper by Kamoun et al. tried to correlate molecular and histological findings to create a consensus molecular classification, finding six major groups in which histology fits partly [49]. These six classes differ in underlying oncogenic mechanisms and tumor microenvironment, showing differences in overall survival, with NE-like having the worst prognosis. In Table 2 we provide an overview of the suggested molecular groups. It is important to underline two major molecular groups based on luminal and basal/squamous expression patterns. In the luminal group tumors show a predominant papillary morphology or stromal infiltration. In the basal/squamous groups, most tumors display squamous differentiation but also tumors with enriched mesenchymal/stromal-like signatures. A very small group (3%) is considered as neuronal/neuroendocrine-like tumors, not all of them corresponding histologically to small or large cell/neuroendocrine morphology.

Nevertheless, reducing the pathological entities to six groups would restrict the precision of pathological diagnosis. Furthermore, some items such as LVI, concomitant CIS, especially if abundant, uni- or multifocal cannot be represented in molecular classifications. Currently, the EAU does not recommend molecular classification for clinical purposes, and as of yet there exists no consensus panel of markers for immunohistochemistry that may be used to perform molecular classification without transcriptomic analysis [4].

### Tumor heterogeneity

One of the major problems in treatment, but also in reporting of urothelial carcinoma, is tumor heterogeneity due to histological subtypes and divergent differentiation as described above. Urothelial carcinoma is known for being extremely variable from one area to the other in the same specimen with high mutational burden. Also, different types of tumor heterogeneity exist: a recent paper of Meeks et al. described the well-known intra-tumor heterogeneity, but also an inter-tumor heterogeneity, which refers to the changes between the primary and the metastatic tumor [54]. Furthermore, they also underlined a temporal heterogeneity, with a tumor changing during its evolution, especially under chemotherapy. Pathology can explore and describe aspects of morphologically observed tumor heterogeneity in biopsy, TURB or cystectomy and lymphadenectomy specimens.

### Targeted therapies with PD-L1 inhibitors

In 2014, Powles et al. provided evidence for the use of immune checkpoint inhibitors (ICI) in urothelial carcinoma and could show that tumors with infiltrating immune cells expressing PD-L1 (programmed death ligand 1) had particularly high response rates to ICI [72]. The American Food and Drug Administration (FDA) and European Medical Agency (EMA) have approved several ICIs as treatment for patients with metastatic and locally advanced UC in specific settings [73]. Since then, ICI have changed the management of UC-patients profoundly and PD-L1 is now the most frequently employed laboratory marker in UC. Five different PD-L1-targeting agents are currently approved for the treatment of locally advanced muscle invasive or metastatic UC.

Each therapy has its own companion test and is only approved for cases with expression of PD-L1 above a defined threshold, using a dedicated antibody for immunolabelling (Table 3): For atezolizumab, the threshold is PD-L1 expression detected in ≥ 5% of immune cells either infiltrating the tumor or within the contiguous peritumoral stroma, using the SP142 antibody clone. For pembrolizumab, the CPS (combined positive score) must be ≥ 10% of tumor and/or immune cells. Several authors claimed that most markers, besides SP142, have overlapping results and are probably interchangeable [74–76]. While immunohistochemistry is rapid and relatively easy to perform, there are several limitations such as interobserver variability and tumor heterogeneity. It is unclear which sample should be tested (TURB, which bloc, cystectomy, metastatic lymph node, distant metastasis). Moreover, some authors demonstrated that PD-L1 is differently expressed in divergent differentiation and subtypes [74, 77].

**Table 3:** PD-L1 companion diagnostic tests

Drug name	Companion test	Cells	Cutoff
Atezolizumab (Tecentriq^©^)	Ventana SP142	IC	≥ 5%
Durvalumab (Imfinzi^©^)	Ventana SP263	IC + TC	≥ 25%
Pembrolizumab (Keytruda^©^)	Dako 22C3	IC, TC (CPS)	CPS ≥ 10
Nivolumab (Opdivo^©^)	Dako 28–8	TC	≥ 5%
Avelumab (Bavencio^©^)	Dako 73–10	TC	≥ 5%

*TC* tumor cells, *IC* immune cells, *CPS* combined positivity score (TC + IC/TC × 100)

It must be underlined that the choice of treatment for patients with metastatic bladder cancer cannot currently be based on any valid predictive biomarker. Different markers such as tumor mutational burden (TMB), molecular subgroups and gene expression signatures have not consistently demonstrated ability to distinguish patient groups for treatment and their use in clinic is discouraged. Expression of PD-L1 in tumor or immune cells assessed by immunohistochemistry appears inconsistently associated with response to checkpoint inhibitors and the use as predictive marker is highly questionable.

### Urine cytology

Since 2015 a new standardized system of cytology reporting, called “The Paris System”, is available and allows to track in a more pertinent way high-grade lesions [78]. This system was encouraged by several organizations such as the College of American Pathologists (CAP) as it focuses on the recognition of high-risk disease and has high sensitivity and specificity in urine specimens. The main categories are listed in Table 4.

**Table 4:** Main categories of The Paris System in cytology reporting

Main categories of The Paris System (TPS)
Non diagnostic/unsatisfactory
Negative for high-grade urothelial carcinoma (NHGUC)
Atypical urothelial cells (AUC)
Suspicious for HGUC (SHGUC)
HGUC
Low-grade urothelial neoplasm (LGUN)
Secondary malignancies

It must be emphasized that a negative report does not mean that tumor presence can be excluded. After 1 year, reported experience of 1814 cases allowed a better categorization of AUC, LGUC and SHGUC [79]. The authors reported significantly fewer low-grade urothelial neoplasms (0.94% vs 1.84%; *P* < 0.05) and more SHGUC cases (2.09% vs 0.73%; *P* < 0.01) compared to before implementation of the Paris System. On the other hand, regarding the HGUC category, neither the frequency (4.69% vs 4.47%) nor the risk of malignancy (89.39% vs 91.04% with HGUC on histology) were found to be significantly different when comparing before and after use of the Paris System.

In case of a positive result and negative cystoscopy, a closer follow-up and eventually repeated biopsies as well as examination of the upper urinary tract should be considered.

The advantage of the Paris System is an internationally uniform approach of urine cytology. All categories are based on well defined and easily reproducible criteria, such as the nuclear-cytoplasmic ratio (> 0.5), severe hyperchromasia, irregular nuclear membranes and/or clumpy chromatin. It must be underlined that the NHGUC group also includes patients with minimal atypia, caused by polyoma viruses, other infections, urolithiasis, chemo- or radiation therapy.

## Conclusion

Continuous international collaborations and exchanges between pathologists, urologists and oncologists have led to standards in the reporting and microscopic diagnosis of BC specimens. Emerging molecular insights already affect our understanding and reporting of BC and are likely to have a greater impact with increasing data and standardization of analysis.

Some areas still lack prospective data (such as prognostic impact of substaging of pT1 disease). Nevertheless, recent progress in reporting and diagnosing to improve patient management has been substantial. Here, we presented an update of pathology reporting intended to aid the clinician in better understand our approach to BC.
